# Supplementary material for: Histone variant H2B.Z acetylation is necessary for maintenance of Toxoplasma gondii biological fitness
Source: bioRxiv. 2023 Feb 24:2023.02.14.528480. Originally published 2023 Feb 14. Preprint. [Version 2] doi: 10.1101/2023.02.14.528480 (PMC9949044; doi:10.1101/2023.02.14.528480)
Supplement: 1 [file NIHPP2023.02.14.528480V2-supplement-1.pdf]

bioRxiv preprint doi: https://doi.org/10.1101/2023.02.14.528480; this version posted February 24, 2023. The copyright holder for this preprint (which was not certified by peer review) is the author/funder, who has granted bioRxiv a license to display the preprint in perpetuity. It is made available under aCC-BY 4.0 International license.

Supplementary figures

Figure S1

bioRxiv preprint doi: https://doi.org/10.1101/2023.02.14.528480; this version posted February 24, 2023. The copyright holder for this preprint (which was not certified by peer review) is the author/funder, who has granted bioRxiv a license to display the preprint in perpetuity. It is made available under aCC-BY 4.0 International license.

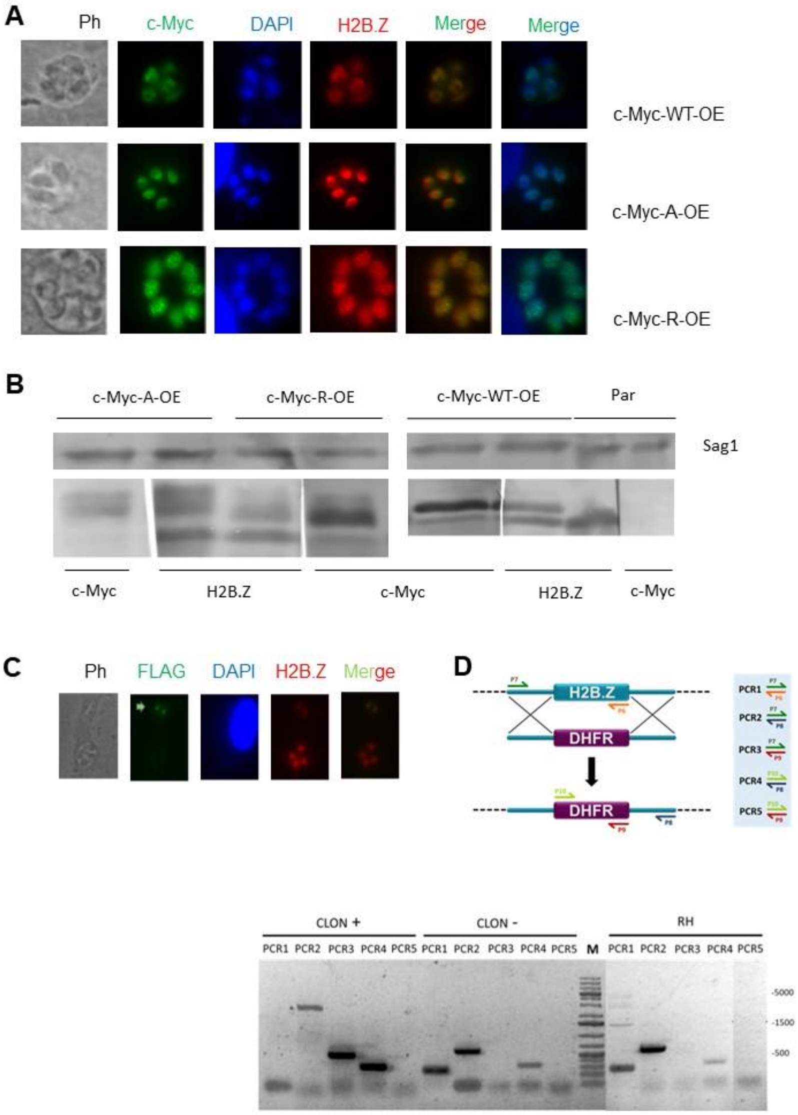

Fig. S1. Controls for tachyzoite lines over-expressing H2B.Z WT or mutants and CRISPR/Cas9 deletion of endogenous H2B.Z gene. A. Immunofluorescence assay. Htert confluent slides were infected with the different clones obtained and immunofluorescence was performed with anti c-Myc (green) and anti-H2B.Z (red); DAPI was used to stain nuclei. Images show a representative IFA of one clone of each over-expressing line obtained. **B.** Western-blot assay. One clone of each line and the parental were lysed and run in SDS-PAGE for WB assay with anti-H2B.Z, anti-c-Myc and anti-Sag1 (charge control). **C.** Immunofluorescence after transfection showing flag-CAS9 signal in the nucleus of one vacuole (green arrow). **D.** PCR selection of positive

bioRxiv preprint doi: https://doi.org/10.1101/2023.02.14.528480; this version posted February 24, 2023. The copyright holder for this preprint (which was not certified by peer review) is the author/funder, who has granted bioRxiv a license to display the preprint in perpetuity. It is made available under aCC-BY 4.0 International license.

clones. The scheme shows the primers designed for PCR detection of positive and negative clones. The image corresponds to a representative PCR where a positive and a negative clone with the parental as control are shown. PCR1-5: primers combinations for PCR shown in the scheme.

Figure S2

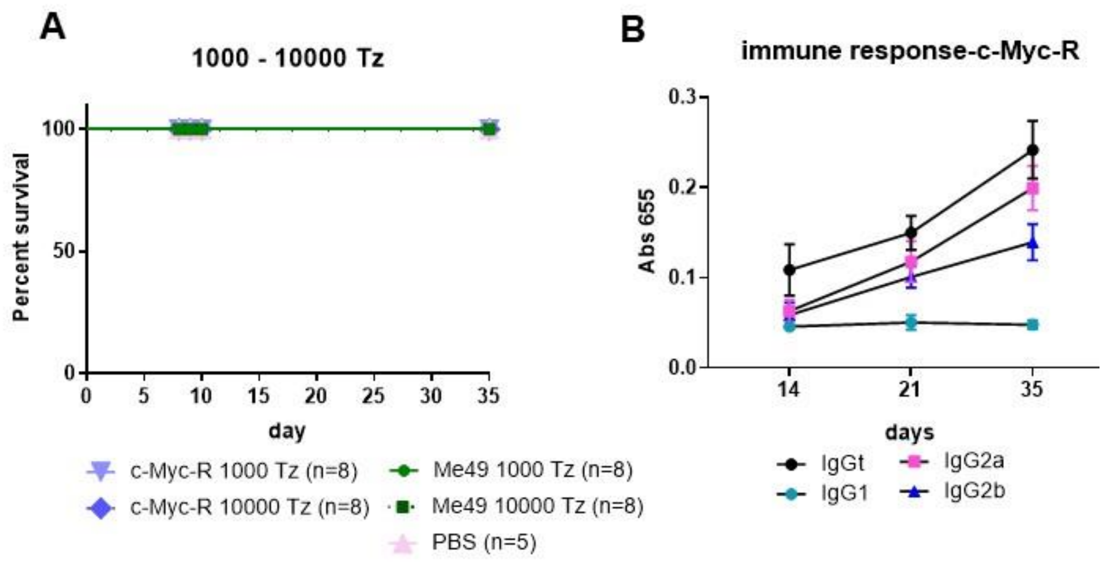

Fig. S2. c-Myc-R tachyzoites up to 10,000 are unable to kill mice but immune response is triggered. A. Survival assay. 10 mice C57BL/6 were intra-peritoneal infected with either 1000 or 10000 tachyzoites of Me49 or c-Myc-R tachyzoites. Vehicle (PBS) was inoculated in 5 mice as control. The survival curve shown corresponds to one assay, representative of three independent experiments. **B.** Evaluation of immune response by ELISA. Assay shown in Fig. 5C, now showing data for 14, 21 and 35 days samples from mice infeceted with c-Myc-R tachyzoites. Total IgG and subtypes (IgG2a, IgG2b and IgG1) were analyzed.

Figure S3

bioRxiv preprint doi: https://doi.org/10.1101/2023.02.14.528480; this version posted February 24, 2023. The copyright holder for this preprint (which was not certified by peer review) is the author/funder, who has granted bioRxiv a license to display the preprint in perpetuity. It is made available under aCC-BY 4.0 International license.

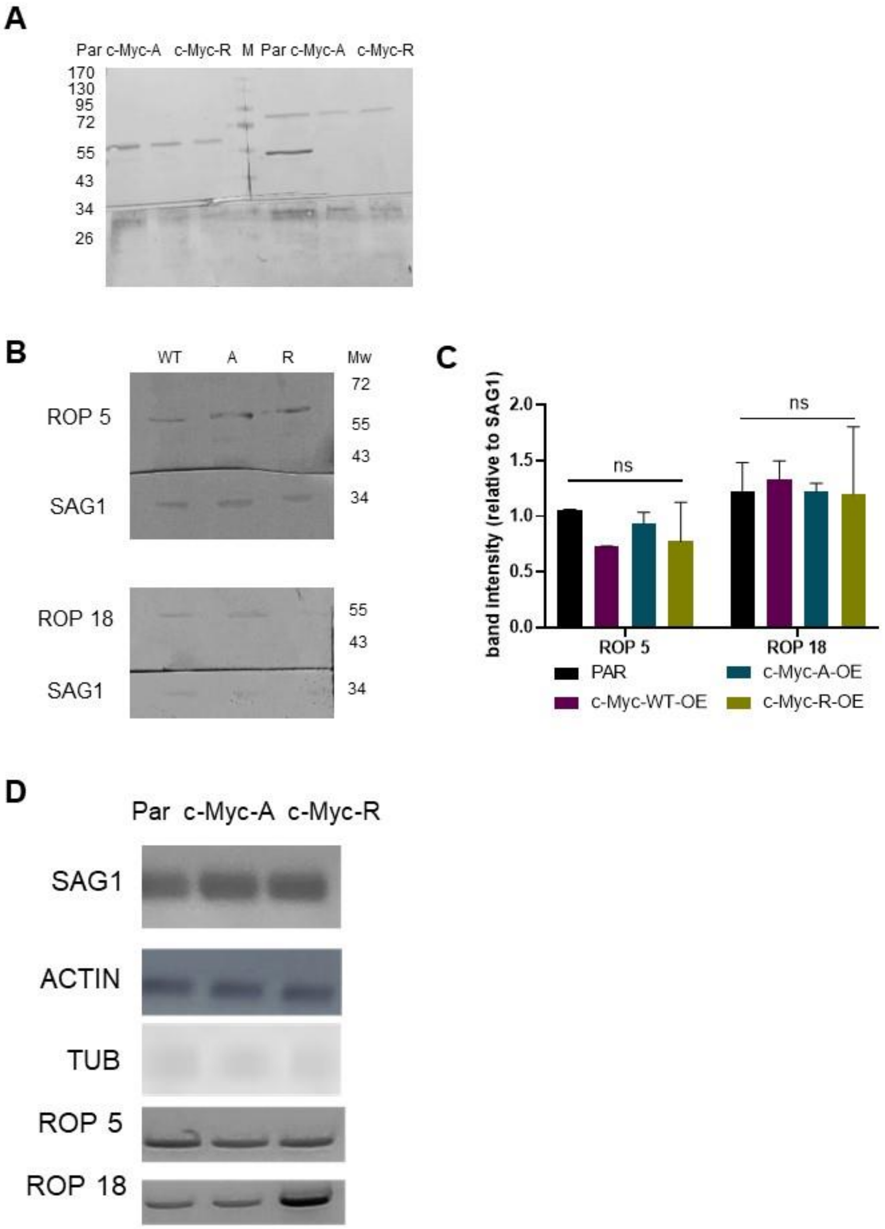

Fig. S3. Western blot analysis of ROP proteins. A. Complete image of Western blot shown in Fig. 5. **B.** Representative Western-blot of over-expressing lines. Antibodies against Rop5, Rop18 and Sag1 were used. WT: c-Myc-WT-OE; A: c-Myc-A-OE; R: c-Myc-R-OE. The upper bands detected with αRop18 are of higher molecular weight than Rop18 full length protein, being unspecific. However, the intensities are similar to Sag1, being a second charge control. **C.** Quantification of Rop5 and Rop18 bands, relativized to Sag1 band intensity in each lane, in two independent WB experiments with the OE lines, and statistical analysis by

bioRxiv preprint doi: https://doi.org/10.1101/2023.02.14.528480; this version posted February 24, 2023. The copyright holder for this preprint (which was not certified by peer review) is the author/funder, who has granted bioRxiv a license to display the preprint in perpetuity. It is made available under aCC-BY 4.0 International license.

GraphPad Prism 8. ns: not significant. **D.** RT-PCR. Tachyzoites of parental (RHΔ*hxgprt*), c-Myc-A and c-Myc-R were collected by triplicate and conserved in TriZol until RNA extraction, and cDNA preparation. PCR was run with primers for the genes indicated and ran in agarose gels. Image is representative of three independent experiments.

Figure S4

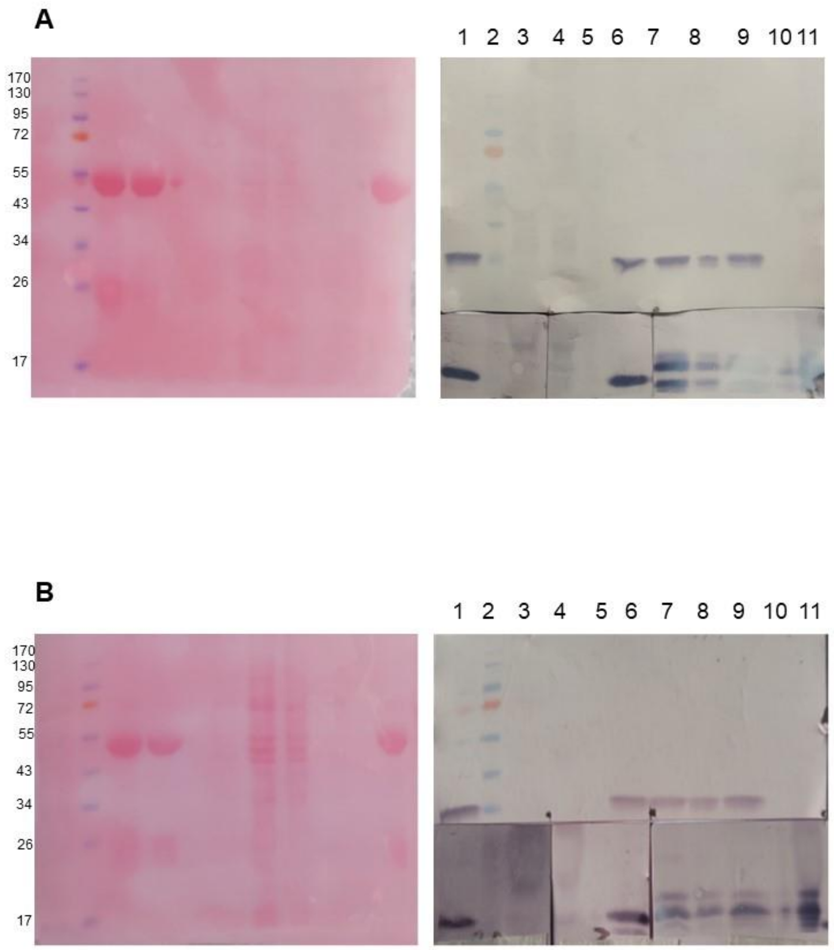

Fig. S4. Co-immunoprecipitation assay. A. Complete image of the Pounceau (left) and WB (right) shown in Fig. 7 for c-Myc-A. **B.** Complete image of the Pounceau (left) and WB (right) shown in Fig. 7 for c-Myc-R. For

bioRxiv preprint doi: https://doi.org/10.1101/2023.02.14.528480; this version posted February 24, 2023. The copyright holder for this preprint (which was not certified by peer review) is the author/funder, who has granted bioRxiv a license to display the preprint in perpetuity. It is made available under aCC-BY 4.0 International license.

both images in lanes 1-11 was run: IN-M-IP-IP-space-IN-IN-T-NU-space-IP. IN: input; IP: immunoprecipitation; T: total; NU: after immunoprecipitation, not bound to agarose beads. MW markers are indicated at the left.
